# Draft Genome Sequence of a Potential Commercial Biocellulose Producer, Strain Komagataeibacter europaeus GH1

**DOI:** 10.1128/MRA.00963-20

**Published:** 2020-10-15

**Authors:** Gulden Nagmetova, Kalysh Berdimuratova, Asylulan Amirgazin, Vladislav Shevtsov, Aisulu Ismailova, Yerlan Ramankulov, Alexandr Shevtsov, Askar Kurmanbayev

**Affiliations:** a National Center for Biotechnology, Nur-Sultan, Kazakhstan; b L. N. Gumilyov Eurasian National University, Nur-Sultan, Kazakhstan; c S. Seifullin Kazakh Agro Technical University, Nur-Sultan, Kazakhstan; d School of Science and Technology, Nazarbayev University, Nur-Sultan, Kazakhstan; Portland State University

## Abstract

In this work, we present the draft genome sequence of Komagataeibacter europaeus strain GH1, which is an extremely efficient biocellulose producer.

## Abstract (precis)

In this work, we present the draft genome sequence of Komagataeibacter europaeus strain GH1 which is an extremely efficient biocellulose producer.

## ANNOUNCEMENT

Acetic acid bacteria (AAB) belong to the *Alphaproteobacteria* family and have a unique metabolic system capable of producing a wide range of saccharides and vitamins and fermenting various biological substrates (1). *Komagataeibacter* spp. have applications in the industrial production of biocellulose for food, biomedicine, cosmetics, and engineering (2). Knowledge of the genomes of industrially valuable strains will increase the productivity of microorganisms and large-scale production.

A detailed description of the isolation of the *Komagataeibacter europaeus* GH1 strain was provided by Nagmetova et al. (3). Accumulated cultures have been identified to the level of the *K. europaeus* species by 16S rRNA gene sequencing using BigDye v3.1 (Applied Biosystems) with primers 8F and 806R (4, 5). The *K. europaeus* GH1 strain is capable of synthesizing a biocellulose film in Hestrin-Schramm (HS) broth with 1% ethanol (pH 4.8 at 30°C) with a peak performance after 7 days of cultivation (3). To determine strain performance, the biofilms were first washed according to the method of Stasiak-Różańska and Ploska (6). The average productivity of biocellulose was 12 g/liter.

DNA was isolated using the DNA minikit (Qiagen). The preparation of libraries was carried out using the Nextera DNA Flex library prep kit (Illumina, USA). Sequencing was performed on a MiSeq system using MiSeq reagent kit v3 (600 cycles). The sequencing platform produced 1,893,358 paired-end reads. The reads were trimmed using Sickle v1.33 (7) up to value Q30 and *de novo* assembled using SPAdes v3.13.2 (8) with a 127 k-mer length in careful mode.

Genome annotation was performed using PGAP v4.11 (9). Determination of functional groups of genes was performed using RAST (10). The search for cellulose synthase operons was carried out using local BLAST+v2.9.0 (11). Identification of prophage sequences was carried out using the PHASTER Web service (12). All software was used with default parameters except when stated otherwise.

The draft genome assembly was obtained with a length of 3,809,040 bp and 105 contigs; the average coverage was 171×, the value of *N*_50_ was 64,110 bp, and the GC content was 61.5%. The annotation predicted 3,352 protein-coding genes and 58 RNA genes. The functional groups involved in carbohydrate and amino acid metabolism are the biggest and contain 187 and 248 genes, respectively. Both types of cellulose synthase operons were identified in the assembly. The bcsI and bcsII operon types are similar to those of the *K. europaeus* SRCM101446, *K. europaeus* 5P3, *K. europaeus* CECT8546, and *K. europaeus* LMG18890 strains but are in contrast to those of the *K. europaeus* LMG18494 and *K. europaeus* NBRC3261 strains, which are characterized by the absence of the bcsII type operon and have two major deletions in the *bcsC* gene (13). An intact prophage located in contig 8 was identified in the genome. This prophage has a length of 40.3 kbp and has 95.65% identity to the prophage in the genome of *K. europaeus* SRCM101446.

### Data availability.

This whole-genome shotgun project has been deposited in DDBJ/EMBL/GenBank under the accession no. NZ_JACHUU000000000. The version described in this paper is the first version, NZ_JACHUU000000000.1. The raw data from BioProject PRJNA649655 were submitted to the NCBI SRA under experiment accession no. SRR12349535.
